# Albumin induces upregulation of matrix metalloproteinase-9 in astrocytes via MAPK and reactive oxygen species-dependent pathways

**DOI:** 10.1186/1742-2094-9-68

**Published:** 2012-04-16

**Authors:** Hantamalala Ralay Ranaivo, Jessica N Hodge, Nicole Choi, Mark S Wainwright

**Affiliations:** 1 Department of Pediatrics, Division of Neurology, Children’s Memorial Hospital, 2300 Children’s Plaza, Chicago, IL, 60614, USA; 2 Department of Pediatrics, Division of Critical Care, Children’s Memorial Hospital, 2300 Children’s Plaza, Chicago, IL, 60614, USA; 3 Center for Interdisciplinary Research in Pediatric Critical Illness and Injury, Northwestern University Feinberg School of Medicine, Chicago, IL, USA

**Keywords:** Matrix metalloproteinase, Astrocyte, Blood brain barrier, Mitogen-activated protein kinases

## Abstract

**Background:**

Astrocytes are an integral component of the blood–brain barrier (BBB) which may be compromised by ischemic or traumatic brain injury. In response to trauma, astrocytes increase expression of the endopeptidase matrix metalloproteinase (MMP)-9. Compromise of the BBB leads to the infiltration of fluid and blood-derived proteins including albumin into the brain parenchyma. Albumin has been previously shown to activate astrocytes and induce the production of inflammatory mediators. The effect of albumin on MMP-9 activation in astrocytes is not known. We investigated the molecular mechanisms underlying the production of MMP-9 by albumin in astrocytes.

**Methods:**

Primary enriched astrocyte cultures were used to investigate the effects of exposure to albumin on the release of MMP-9. MMP-9 expression was analyzed by zymography. The involvement of mitogen-activated protein kinase (MAPK), reactive oxygen species (ROS) and the TGF-β receptor-dependent pathways were investigated using pharmacological inhibitors. The production of ROS was observed by dichlorodihydrofluorescein diacetate fluorescence. The level of the MMP-9 inhibitor tissue inhibitor of metalloproteinase (TIMP)-1 produced by astrocytes was measured by ELISA.

**Results:**

We found that albumin induces a time-dependent release of MMP-9 via the activation of p38 MAPK and extracellular signal regulated kinase, but not Jun kinase. Albumin-induced MMP-9 production also involves ROS production upstream of the MAPK pathways. However, albumin-induced increase in MMP-9 is independent of the TGF-β receptor, previously described as a receptor for albumin. Albumin also induces an increase in TIMP-1 via an undetermined mechanism.

**Conclusions:**

These results link albumin (acting through ROS and the p38 MAPK) to the activation of MMP-9 in astrocytes. Numerous studies identify a role for MMP-9 in the mechanisms of compromise of the BBB, epileptogenesis, or synaptic remodeling after ischemia or traumatic brain injury. The increase in MMP-9 produced by albumin further implicates both astrocytes and albumin in the acute and long-term complications of acute CNS insults, including cerebral edema and epilepsy.

## Background

The blood–brain barrier (BBB) is composed of vascular endothelium, basal lamina, pericytes and astrocyte foot processes anchored by tight junctions [1-3]. The BBB prevents fluid, macromolecules, and small molecules from exiting the microvasculature and entering the brain parenchyma. Compromise of the BBB by ischemic or traumatic brain injury results in cytotoxic and vasogenic edema, and is a major determinant of outcome after neurological trauma [4-7].

The endopeptidase matrix metalloproteinase (MMP)-9 plays a pivotal role in BBB proteolysis after injury [8-10], and contributes to cell death after prolonged seizures [11]. MMP-9 degrades tight-junction proteins [12], regulates N-methyl-D-aspartate (NMDA) receptor signaling [3,13] and synaptic remodeling [3], also implicating this proteinase in the mechanisms of long-term potentiation and epileptogenesis [14]. Under normal conditions, the proteolytic activity of MMPs including MMP-9 is regulated by tissue inhibitor of matrix metalloproteinase (TIMP)-1. Gene transfer and knockout approaches indicate a protective role for TIMP-1 after cerebral ischemic insults [15,16].

Endothelial cells are known to be the principal structural component of the BBB, but relatively less is known about the function of astrocytes in the mechanisms leading to compromise of the BBB after injury. Astrocytes play a major role in maintaining water homeostasis and integrity of BBB under physiological and pathophysiological conditions [17]. MMP-9 activation in astrocytes can by induced by oxidative stress [18], thrombin [19], tumor necrosis factor-α [20], or tissue plasminogen activator [21], and involves activation of mitogen-activated protein kinases (MAPKs) [19,20].

Following disruption of the BBB, blood-derived proteins including thrombin and albumin, penetrate into the brain parenchyma. Albumin is taken up by astrocytes [22,23] and can then initiate a cascade of events implicated in the mechanisms of epileptogenesis via activation of the transforming growth factor (TGF-β) receptor [22,24]. Albumin also activates intracellular calcium signaling pathways in astrocytes [25], and causes the release of inflammatory factors including monocyte chemotactic protein (MCP)-1 [26], interleukin (IL)-1β, nitric oxide and chemokine (C-X3-C motif) ligand (CX3CL)1 [27,28]. We have previously shown that activation of astrocytes by albumin involves MAPK pathways [27-29]. The effects of albumin on astrocyte expression of MMP-9, and thereby the potential role of albumin in the mechanisms leading to brain edema, are unknown.

Understanding the complex effects of albumin and other serum proteins on glial responses to acute brain injuries has important implications for clinical practice. Animal models of traumatic brain injury [30], intracortical hematoma [31], and stroke [32] indicate a neuroprotective role for albumin. The increase in mortality associated with albumin treatment after traumatic brain injury (TBI) [33] contrasts with the improved functional outcome seen 2 years after ischemic stroke [34].

In this study, we investigated whether albumin activates the production of MMP-9 by astrocytes. We examined the involvement of MAPK pathways including p38 MAPK, extracellular signal regulated protein kinase (ERK) and c-Jun N-terminal kinase (JNK) in these responses. We also determined the role of reactive oxygen species (ROS) and the role of TGF-β receptor pathway in the production of MMP-9 induced by albumin in astrocytes. We found that albumin induces an increase in the level of MMP-9 and that this increase in MMP-9 is dependent on the activation of MAPK pathways and ROS. These findings implicate albumin in the mechanisms of cerebral edema and epileptogenesis after brain injury.

## Methods

All experiments followed protocols approved by the Institutional Animal Care and Use Committee of Children’s Memorial Research Center, Chicago, Illinois.

### Isolation and culture of primary astrocytes

Primary cortical astrocyte cultures were prepared from Sprague-Dawley rat pups 1–3 days old (Charles River, Wilmington, MA, USA), as described previously [29,35]. Briefly, cortices were isolated and cleaned of meninges in Ca^2+^ and Mg^2+^-free Hank’s balanced buffered salt solution (HBSS). After trypsin digestion, the cell suspension was filtered through a 40 μm filter, separated by centrifugation, and resuspended in DMEM supplemented with 10% FBS and 1% penicillin and streptomycin. Cells were then transferred to 75 cm^2^ flasks, and cultured in humidified incubator at 37°C in 5% CO_2_, with media changed every 2 to 3 days. After 9 to 10 days in culture, enriched astrocyte cultures were prepared by shaking the flasks at 200 rpm for 24 hours, and the media containing floating microglia cells and oligodendrocytes then removed and replaced. When confluent, cells were lifted from the flask with 0.05% trypsin/0.2% EDTA and plated into 12-well plates. Cells were cultured to confluency in humidified incubator at 37°C in 5% CO_2_ with the media changed every 3 to 4 days. The enriched astrocyte cultures were composed of more than 95% of astrocytes, as determined by staining using an anti-glial fibrillary acidic protein as the primary antibody and the nuclear staining dye DAPI as previously described [28] (results not shown).

### Astrocyte activation with albumin

Culture media was changed to serum-free, phenol red-free DMEM supplemented with 1% N2 supplement (Gibco/Invitrogen, Carlsbad, CA, USA) 24 hours before treatment. Cells were treated with either PBS (control) or 0.1 mmol/l globulin-free and fatty acid-free BSA (Sigma Aldrich, St. Louis, MO, USA). The concentration of BSA used in this study is similar to that used in other studies of the effects of exogenous albumin on primary astrocytes or brain slice preparations [22,24], and corresponds to 25% of the serum concentration. We have previously reported that this concentration of BSA does not induce any change in cell viability [28].

### Pharmacologic inhibition of mitogen-activated protein kinases, transforming growth factor-β receptor signaling, and reactive oxygen species

Cells were treated with inhibitors of the MAPK pathways: the p38 MAPK inhibitor SB203580, the MAPK kinase (MEK)/ERK pathway inhibitor PD98059, JNK inhibitor SP600125 (Calbiochem, Gibbstown, NJ, USA). The role of the TGF-β receptor pathway was investigated by treating the cells with the TGF-β receptor I inhibitor SB431542 (Tocris, Ellisville, MO, USA) or the specific Smad3 inhibitor (SIS3, Calbiochem, San Diego, CA, USA). The role of ROS was determined by treating the cells with the NADPH oxidase inhibitor diphenyleneiodonium chloride (DPI), polyethylene glycol–superoxide dismutase (PEG-SOD, 200U), or polyethylene glycol–catalase (PEG- CAT, 200U) (all Sigma Aldrich). For all conditions, the inhibitor or diluent was added to the cells 30 minutes before treatment with PBS or BSA.

### Quantification of mitogen-activated protein kinase activation by western blotting

Cells lysates were prepared as described previously [27,28,35]. Equal amounts of protein were determined by the bicinchoninic acid protein assay (Pierce, Rockford, IL, USA). Samples were added to 5× Laemmli sample buffer, heated at 90°C for 5 minutes, then separated in a 10% gel (Mini-Protean TGX) and transferred to a polyvinylidene fluoride membrane (all Bio-Rad, Hercules, CA, USA). Membranes were blocked with Tris-buffered saline containing 0.1% Tween-20 and 5% non-fat dry milk for 1 hour at room temperature. Membranes were then incubated overnight at 4°C with either anti-phospho-p38 MAPK, anti-phospho-ERK1/2 or anti-phospho-JNK (all Cell Signaling Technology, Danvers, MA, USA), followed by incubation with horseradish perodixase-conjugated secondary antibodies for 1 hour at room temperature. A chemiluminescent substrate was used to detect signals. To measure the expression of the total MAPK proteins, membranes were incubated with antibodies to total p38 MAPK, ERK1/2, and JNK respectively (all Cell Signaling Technology). Autoradiography films were scanned and analyzed for relative densitometry with Molecular Imaging software (version 5.0.2.30; Carestream, Rochester NY). Ratios of phospho- to total p38 MAPK, ERK1/2, or JNK were calculated, and data were normalized using the control group or the BSA-treated group as 100%.

### Gelatin zymography

Conditioned media underwent a purification step before being used in a zymography assay as described previously [35]. Samples were resolved by electrophoresis in a 10% polyacrylamide gel containing gelatin (Invitrogen). Thereafter, gels were washed four times in renaturing buffer (Invitrogen) for 15 min each before incubating for 16 hr at 37°C in development buffer (Invitrogen). After staining the gel with 0.1% Coomassie Brilliant Blue R-250 (Bio-Rad), the gelatinolytic activities were visualized as a clear band in the uniformly stained background. The molecular weight of the gelatinase was estimated by comparing the migration distance of the clear bands with the distance migrated by markers of known molecular weight. The gels were scanned using white light transillumination in an imaging system (Gel Logic 212 PRO; Carestream). The bands were analyzed for relative densitometry using the Molecular Imaging software (Carestream).

### Detection of intracellular reactive oxygen species production

Cells were treated with PBS, BSA, or 100 μmol/l of the positive control *tert*-butyl hydroperoxide (TBHP; Invitrogen) for 90 min. The fluorogenic marker 5-(and-6)-carboxy-2′,7′-dichlorodihydrofluorescein diacetate (carboxy-H_2_DCF-DA, Invitrogen) was used to monitor the intracellular production of ROS. Cells were washed with HBSS and incubated for 30 minutes with HBSS containing 25 μmol/l carboxy-H_2_DCF-DA at 37°C. Cell nuclei were stained using Hoescht 33342 (Invitrogen). Cells were washed with HBSS and visualized using an inverted microscope (DM-IRB; Leica, Heerbrugg, Switzerland) coupled with a camera (Retiga 4000R; QImaging, Surrey, BC, Canada), and fluorescence images were acquired using a fluorescence camera (ORCA ER; Hamamatsu. Bridgewater, NJ, USA) camera and pseudocolored using OpenLab 5.5 (Improvision, Waltham, MA, USA). The fluorescence signal was assessed qualitatively.

### ELISA

Levels of TIMP-1 in the cell culture media were measured by ELISA (R & D Systems, Minneapolis, MN, USA) according to the manufacturer’s instructions.

### Statistical analysis

Data are expressed as mean ± SEM. Time-course analysis was performed using two-way repeated analysis of variance (ANOVA). Comparisons between multiple groups were performed with ANOVA followed by Dunnett’s multiple comparison, comparing all the groups to the BSA-treated group. The criterion for statistical significance was *P* < 0.05. GraphPad Prism (version 5.0; GraphPad Software, Inc., San Diego, CA, USA) was used for statistical analyses.

## Results

### Bovine serum albumin produces a time-dependent increase in levels of MMP-9

**Figure 1:**
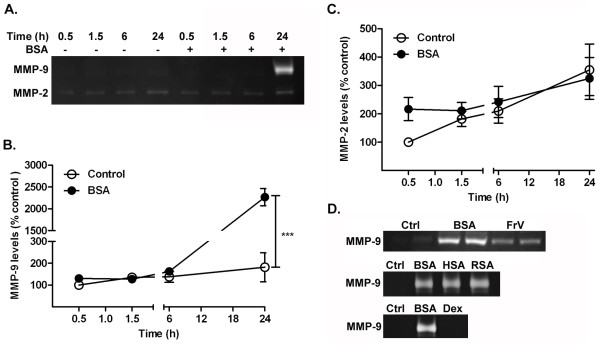
**Albumin stimulated an increase in the release of MMP-9 released in the conditioned media by astrocytes.** Astrocytes were exposed to control PBS (−) or BSA (+). The conditioned media were collected after 0.5, 1.5, 6 and 24 hours and analyzed by gelatin zymography. **(a)** Representative zymogram showing a time-dependent increase in matrix metalloproteinase (MMP)-9; **(b)** there was a significant increase in the level of MMP-9 released 24 hours after BSA treatment compared with the control group; **(c)** the level of MMP-2 measured after BSA treatment was similar to that of the control group at all time points; **(d**) treatment with fraction V (FrV) BSA also induced an increase in the level of MMP-9 released from astrocytes. Human serum albumin (HSA) and rat serum albumin (RSA) induced a similar increase in MMP-9 level to that seen with BSA. Treatment with dextran (0.1 mmol/L) did not induce any change in the level of MMP-9 in astrocytes. Data are representative of mean ± SEM of three independent experiments. ***P < 0.001 compared with control group, by two-way ANOVA.

Using zymography, we determined the effect of albumin on the MMP-9 levels released in the conditioned media at different time points (Figure 1). The release of MMP-9 from astrocytes treated with albumin was time-dependent. The increase in MMP-9 was detected at 24 hours after exposure to albumin, and was significantly increased compared with control cells (Figure 1a,b). No MMP-9 was detected in control media at any of the time points investigated. MMP-2-related gelatinase activity was detected in control media at all the time points studied. Treatment of astrocytes with albumin did not affect the levels of MMP-2 in media compared with control values (Figure 1c).

We then investigated whether the increase in MMP-9 was specific to the type of albumin (globulin- and fatty acid-free BSA) and the species used in these experiments. We treated astrocytes with the same concentration of either the BSA used above, or the fraction V (FrV) preparation, which still contains fatty acids. We measured the release of MMP-9 after 24 hours by zymogram. Treatment with FrV and BSA both produced an increase in MMP-9 compared with control cells (Figure 1d). Both rat serum albumin and human serum albumin induced an increase in MMP-9 that was similar to that produced by BSA. Thus, the increase in MMP-9 seen in astrocytes was also not dependent on the species of origin of the albumin. None of the albumin preparations tested above induced a change in the level of MMP-2 produced by astrocytes (data not shown). Finally, we examined whether the response to BSA was specific by comparing it with the response to another high molecular weight molecule. Cells treated with 0.1 mmol/l dextran (70 kDa) did not show any increase in the level of MMP-9 compared with control cells (Figure 1d), and dextran did not induce any change in the level of MMP-2 produced by astrocytes (data not shown).

**Figure 2:**
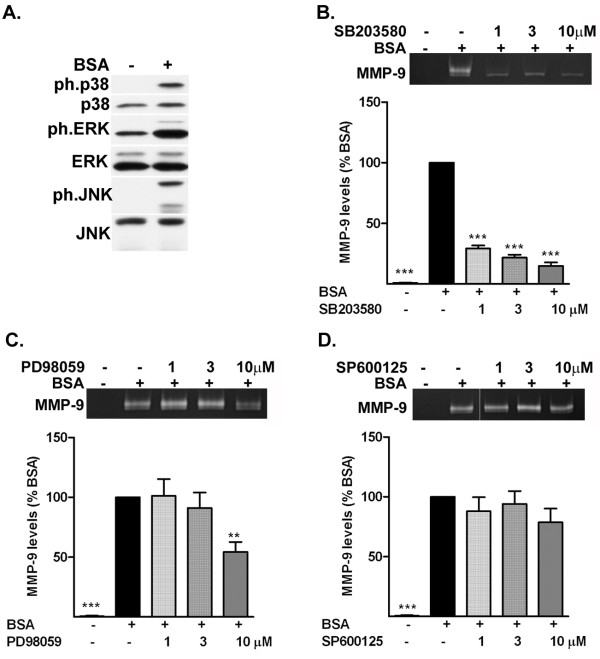
**Albumin-induced increase in matrix metalloproteinase (MMP)-9 is dependent on the activation of mitogen-activated protein kinase (MAPK) pathways. (a)** Representative western blot shows that treatment with BSA for 90 minutes induced an increase in the level of phosphorylated p38 MAPK, extracellular signal regulated protein kinase (ERK) and c-Jun N-terminal kinase (JNK). Representative zymogram and corresponding quantification of the level of MMP-9 release by astrocytes treated with albumin in the presence (+) or absence (−) of **(b)** the p38 MAPK inhibitor SB203580, **(c)** the ERK pathway inhibitor PD98059, and **(d)** the JNK inhibitor SP600125. Inhibition of the p38 MAPK and ERK pathways significantly suppressed the release of MMP-9 induced by albumin. whereas inhibition of JNK pathway did not modify the response to albumin. Data are representative of mean ± SEM of three independent experiments. **P < 0.01, ***P < 0.001 compared with the BSA-treated group.

### Albumin-induced increase in matrix metalloproteinase-9 is suppressed by inhibition of p38 mitogen-activated protein kinase and extracellular signal regulated protein kinase, but not c-Jun N-terminal kinase

We have previously shown that activation of astrocytes induced by albumin involves activation of the MAPK pathways [28]. We confirmed this finding here by showing that treatment of the astrocytes with albumin for 90 minutes induced an increase in the level of phosphorylated p38 MAPK, ERK and JNK (Figure 2a).

To determine whether the activation of MMP-9 produced by albumin was mediated by MAPKs, we pretreated astrocytes with either the p38 MAPK inhibitor SB203580, the ERK pathway inhibitor PD98059, or the JNK inhibitor SP600125. We then exposed the cells to albumin and measured MMP-9 activation after 24 hours of recovery (Figure 2b-d). Inhibition of the p38 MAPK pathway significantly attenuated the increase in MMP-9 induced by albumin (Figure 2b). Inhibition of the ERK pathway significantly attenuated the increase in MMP-9 induced by albumin, but only at the highest concentration of inhibitor used (Figure 2c). In contrast, the release of MMP-9 in response to albumin was not affected by inhibition of JNK (Figure 2d). The level of MMP-2 measured in the conditioned media of astrocytes was not affected by the presence of the MAPK inhibitors (data not shown).

### Albumin-induced increase in matrix metalloproteinase-9 is mediated via NADPH oxidase and reactive oxygen species

**Figure 3:**
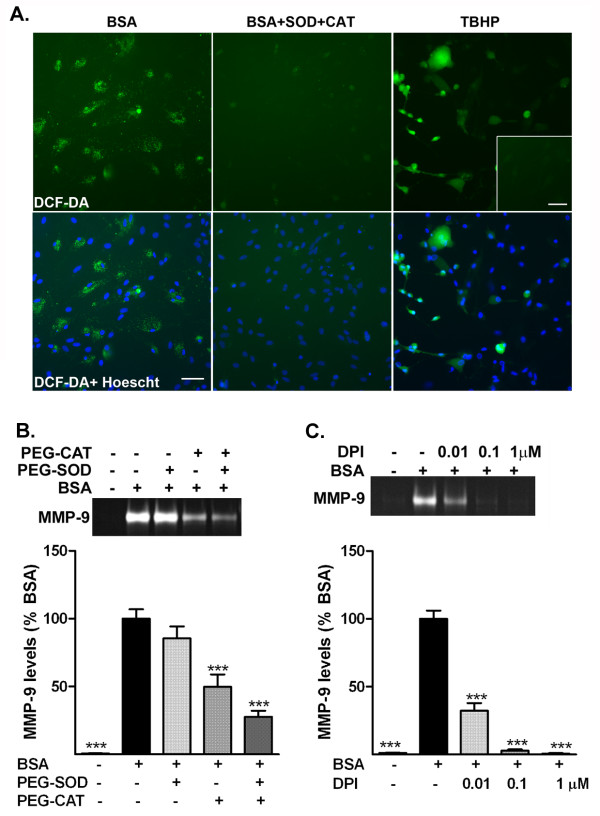
**Albumin-induced increase in matrix metalloproteinase (MMP)-9 involves reactive oxygen species. (a)** Representative images of 5-(and-6)-carboxy-2′,7′-dichlorodihydrofluorescein diacetate (carboxy-H_2_DCF-DA) fluorescence alone (green) or merged with DAPI nuclear stain (blue) in astrocytes treated with BSA, PBS (inset) or BSA with polyethylene glycol–superoxide dismutase (PEG-SOD, 200U) or polyethylene glycol–catalase (PEG-CAT) or the positive control *tert*-butyl hydroperoxide (TBHP). Treatment with albumin induced an increase in the DCF-DA fluorescence in astrocytes that was suppressed by the presence of PEG-SOD and PEG-CAT. Bars represent 50 μm. The data represent two independent experiments, each performed in duplicate. Representative zymogram and corresponding quantification of the level of MMP-9 release by astrocytes treated with albumin in the presence (+) or absence (−) of **(b)** PEG-SOD and PEG-CAT, and **(c)** the NADPH oxidase inhibitor, diphenyleneiodonium chloride (DPI). Inhibition of albumin-induced increase in MMP-9 by antioxidant enzymes SOD and CAT and the NADPH oxidase inhibitor, DPI suggest that the increase in MMP-9 involves reactive oxygen species. Data are representative of mean ± SEM of three independent experiments. ***P < 0.001 compared with the BSA-treated group.

Treatment of astrocytes with albumin induced an increase in the production of ROS as measured by increase in carboxy-H_2_DCF-DA fluorescence (Figure 3a) compared with the control cells (inset, Figure 3a). Pre-treatment of cells with a combination of the antioxidant enzymes PEG-SOD and PEG-CAT suppressed the DCF-DA fluorescence caused by albumin, indicating that the fluorescent signal was due to an increase in ROS. Exposure to the positive control, TBHP, confirmed that increased DCF-DA fluorescence can be detected in astrocytes in the presence of oxidative stress.

Treatment with PEG-CAT alone, or in combination with PEG-SOD, significantly suppressed the MMP-9 production induced by albumin (Figure 3b). However, pre-treatment with PEG-SOD alone did not induce a significant change in the level of MMP-9 produced by astrocytes.

Next, we determined the role of NADPH oxidase in albumin–induced production of MMP-9 by treating the cells with the NADPH oxidase inhibitor, DPI (Figure 3c). The increase in MMP-9 level induced by albumin treatment was significantly suppressed by DPI. Taken together, these data suggest that ROS produced by NADPH oxidase in astrocytes probably mediate the production of MMP-9 by albumin in astrocytes. Neither of these inhibitors induced a change in the level of MMP-2 produced by astrocytes (data not shown).

### Albumin-induced increase in p38 mitogen-activated protein kinase and Jun kinase is downstream from activation of NADPH oxidase

**Figure 4:**
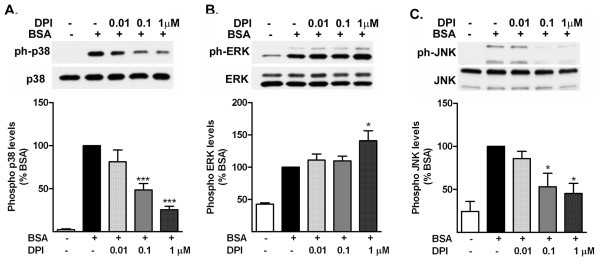
**Albumin-induced increase in p38 mitogen-activated protein kinase (MAPK) and Jun kinase (JNK) is mediated by reactive oxygen species.** Representative pictures and corresponding quantification of western blot of the level of phosphorylated **(a)** p38 MAPK, **(b)** extracellular signal regulated protein kinase (ERK), and **(c)** JNK in cells treated with vehicle or BSA in the presence (+) or absence (−) of the NADPH oxidase inhibitor, diphenyleneiodonium chloride (DPI). Treatment with DPI suppresses the increase in phospho-p38 MAPK and JNK but induces a further increase in the level of phospho-ERK induced by albumin treatment. The data are representative of mean ± SEM of two independent experiments. * *P* < 0.05, ***P < 0.001 compared with the BSA-treated group.

Next, we investigated whether the activation of MAPKs by albumin was dependent on the production of ROS (Figure 4). Inhibition of NADPH oxidase with DPI suppressed the increase in the levels of phospho-p38 MAPK induced by albumin treatment (Figure 4a). Treatment of the astrocytes with DPI induced an increase in the level of phospho-ERK measured in the astrocytes at the highest concentration (Figure 4b). DPI suppressed the increase in the levels of phospho-JNK induced by albumin treatment (Figure 4c).

### Albumin-induced increase in matrix metalloproteinase-9 does not involve the transforming growth factor-β receptor pathway

**Figure 5:**
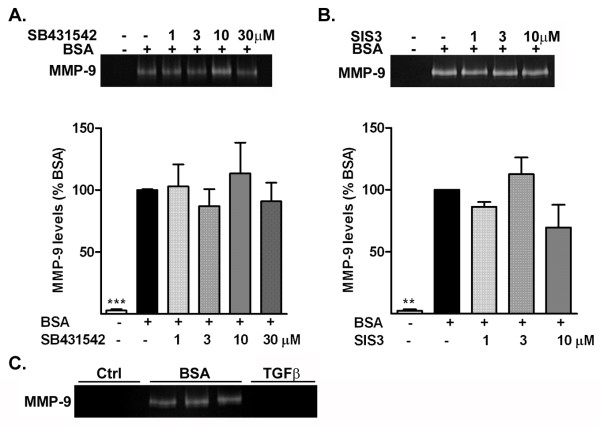
**Albumin-induced increase in matrix metalloproteinase (MMP)-9 occurs independently of the transforming growth factor (TGF)-β receptor.** Representative zymogram and corresponding quantification of the level of MMP-9 release by astrocytes treated with BSA in the presence (+) or absence (−) of **(a)** the TGF-β receptor I inhibitor SB431542, or **(b)** the Smad pathway inhibitor SIS3; neither inhibition of the TGF-β receptor I inhibitor nor the Smad pathway inhibited albumin-induced increase in MMP-9. **(c)** Representative zymogram of the level of MMP-9 release by astrocytes treated with BSA and TGF-β1 (10 ng/mL). Data are representative of mean ± SEM of three independent experiments. ***P < 0.001 compared with the BSA-treated group.

The TGF-β receptor has been previously shown to act as a receptor for albumin on astrocytes [22,24]. We previously showed that the effect of albumin on astrocyte activation partially involves the TGF-β receptor pathway, including activation of the canonical Smad signaling pathway [27]. Accordingly, we next investigated whether the effects of albumin on MMP-9 production also involved the TGF-β receptor pathway (Figure 5). Inhibition of the TGF-β receptor I with SB431542 did not affect the increase in MMP-9 induced by albumin (Figure 5a). Similarly, inhibition of the Smad pathway with SIS3 did not suppress the increase in MMP-9 produced by the albumin-treated astrocytes (Figure 5b). Consistent with these data, treatment of astrocytes with TGF-β1 (10 ng/mL) did not alter the level of MMP-9 in astrocytes (Figure 5c). These data suggest that the increase in MMP-9 induced by albumin in astrocytes occurs independently of the TGF-β receptor and the Smad pathway.

### Albumin induces an increase in tissue inhibitor of metalloproteinase-1 production independent of mitogen-activated protein kinase pathways

**Figure 6:**
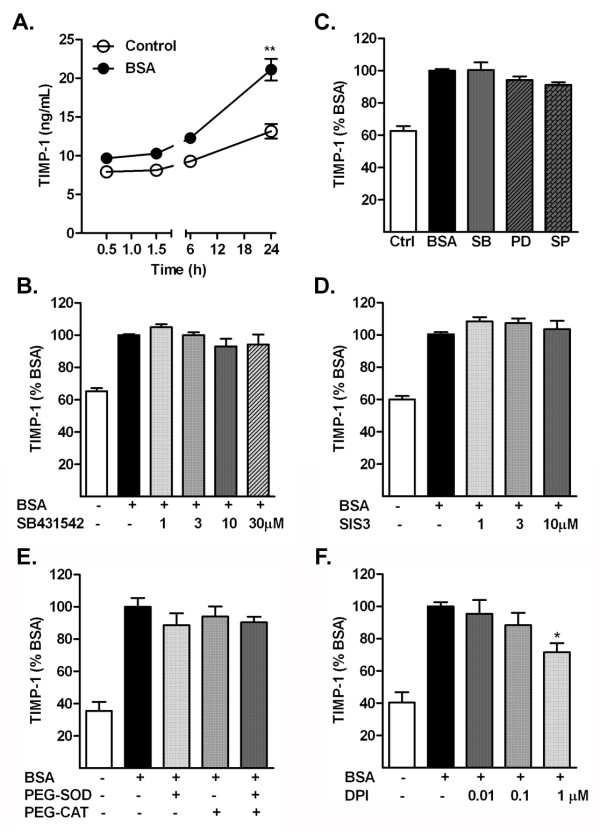
**Albumin stimulates an increase in the release of tissue inhibitor of metalloproteinase (TIMP)-1 in the conditioned media by astrocytes. (a)** Astrocytes exposed to BSA had a time-dependent increase in TIMP-1. **(b)** Levels of TIMP-1 in astrocytes treated with albumin in the presence (+) or absence (−) of the TGF-β receptor I inhibitor SB431542. **(c)** Levels in astrocytes treated with albumin in the presence of the p38 mitogen-activated protein kinase (MAPK) inhibitor SB203580 (SB), the extracellular signal regulated protein kinase (ERK) pathway inhibitor PD98059 (PD), and the c-Jun N-terminal kinase (JNK) inhibitor SP600125 (SP). **(d)** Levels in astrocytes treated with albumin in the presence (+) or absence (−) of SIS3, the Smad pathway inhibitor. **(e)** Levels of TIMP-1 **in** astrocytes treated with albumin in the presence (+) or absence (−) of the antioxidant enzymes superoxide dismutase (SOD) and catalase (CAT) or **(f)** the NADPH oxidase inhibitor diphenyleneiodonium chloride (DPI). Data are representative of mean ± SEM of 3–4 independent experiments, and are expressed (B-F) as a percentage of the value of the BSA-treated group. **P < 0.01 compared with control-treated group. *P < 0.05 compared with BSA-treated group.

Treatment of astrocytes with albumin also induced the production of endogenous inhibitor of MMP-9, TIMP-1 (Figure 6). The time course of expression of TIMP-1 after exposure to albumin was similar to activation of MMP-9, with the maximum level reached at 24 hours (Figure 6a). The level of TIMP-1 also increased over time in the control group but was significantly lower than the albumin-exposed group. The increase in TIMP-1 was not suppressed by inhibition of the p38 MAPK, ERK or JNK pathways (Figure 6b). Furthermore, inhibition of TGF-β receptor I (Figure 6c) or the Smad pathway (Figure 6d) did not suppress the increase in TIMP-1 induced by exposure to albumin. Finally, inhibition of ROS generation by treatment with SOD and CAT (Figure 6e) did not suppress the increase in TIMP-1, and inhibition of NADPH oxidase by DPI (Figure 6f) only partially suppressed TIMP-1 increase at the highest concentration used.

## Discussion

In this study, we found that albumin induces an increase in the production of MMP-9 in astrocytes, and that this increase requires generation of ROS and activation of the MAPK pathway. These findings identify albumin as another signaling molecule in addition to thrombin [19,36], which can activate MMP-9 in astrocytes. These results link albumin to the diverse cellular responses mediated by MMP-9, including neuronal injury, intracerebral hemorrhage, epileptogenesis, and dendritic remodeling [2,3,10,37].

The implications of these data for the use of albumin in cerebral injury in clinical practice are complex. The Saline vs. Albumin Fluid Evaluation (SAFE) study identified a significantly higher mortality rate in patients with severe TBI assigned to albumin compared with the saline group [33]. In stroke subjects, the Albumin in Acute Stroke (ALIAS) Trial showed a potential beneficial therapeutic effect for albumin [34], and the second part of the ALIAS trial has been started with more stringent exclusion criteria [38]. By contrast, pre-clinical [30,39,40] and clinical [41] data indicate improved neurologic outcomes in patients with stroke who were treated with albumin [42].

The contrasting effects of albumin in stroke and TBI reflect the complexity of the cellular responses to MMP-9. Activation of MMPs results in the degradation of the components of the vascular basement membrane, leading to breakdown of the BBB. MMP-9 levels increase after acute brain injuries including status epilepticus [11], and are linked to increases in permeability of the BBB [10,12,43-45]. In a mouse global cerebral ischemia model, neurologic injury was reduced in MMP-9 knockout mice, in part due to attenuated proteolysis of the BBB [46]. Deletion of MMP-9 [47,48], or inhibition of MMP-9 activity [44,47,49], improved neurologic function after TBI or stroke. Furthermore, suppression of the increase in MMPs produced by closed head injury [50,51] or by stroke [52] results in a reduction of the brain edema and improved neurological recovery. However, other lines of evidence implicate MMP-9 in the mechanisms of epileptogenesis and synaptic remodeling [53]. MMP-9 KO mice show increased resistance to pentylenetetrazole kindling–induced epilepsy [14]. Acting through an integrin β1-dependent pathway, MMP-9 produces changes in dendritic spine morphology [2,3]. Our finding that albumin increases MMP-9 activation in astrocytes suggests another pathway linking albumin to the mechanisms of epileptogenesis mediated by the TGF-β receptor [22,24].

The link between activation of p38 MAPK, ERK and MMP-9 found in the present study is consistent with the well-established role for MAPK pathways in the production of MMP-9 in astrocytes in response to different stimuli [20,35,54,55]. Our data indicate a predominant requirement for activation of p38 MAPK. By contrast, the activation of MMP-9 produced by exposure to thrombin acting via protease-activated receptor 1, or stimulation of protein kinase C, is regulated by ERK1/2 [19,20]. This suggests that the specific MAPK involved in signal transduction to MMP-9 will depend on the inciting stimulus. We speculate that the delay in production of MMP-9 in astrocytes in response to albumin reflects *de novo* protein synthesis through transcription and translation, as shown previously in response to IL-1β [54], which takes several hours. However, we cannot exclude the possibility that MMP-9 increases earlier than 24 hours after injury.

The proteolytic activity of MMPs including MMP-9 is regulated by TIMP-1 [15], and we found an increase in TIMP-1 levels over time both in control and albumin-exposed astrocytes. The concomitant changes in expression of metalloproteinases and their endogenous inhibitors have been described in TBI [56] and in ischemia [57], consistent with the increase in TIMP-1 we observed in response to albumin. Evidence from other disease states including experimental autoimmune encephalomyelitis and spinal cord injury suggests that expression of TIMP-1 increases along with MMPs [58,59]. The *in vivo* effects of the increase in MMP-9 may therefore be determined by its activity relative to TIMP-1, as has been suggested for the use of MMP-9 as a clinical biomarker in stroke [10].

The effects of albumin on astrocytes have been reported to involve its binding to surface proteins that act as receptors [22,60]. Our data suggest that the increase in MMP-9 induced by albumin occurs independently of the TGF-β receptor–Smad pathway. We also found that treatment of the cells with TGF-β1 did not increase the level of MMP-9. Consistent with this result, we have previously shown that exposure of astrocytes to TGF-β1 did not alter levels of other inflammatory markers in astrocytes [27]. By contrast, treatment of an astrocyte cell line and primary astrocyte cultures with TGF-β1 has been reported to produce an increase in MMP-9 [61]. The dose of TGF-β1 used in the present study is lower (10 ng/ml) than that used (15 ng/ml) by Hsieh and colleagues, which may account for the difference in the responses.

We found that an increase ROS was required for activation of MMP-9 induced by albumin. This is consistent with previous reports showing that ROS are involved in the production of MMP-9 by astrocytes in response to other stimuli, including IL-1β [62], TGF-β [61], and hemoglobin [18].

The effects of albumin on other components of the neurovascular unit, including endothelial cells, are not well understood. In endothelial cells, oxidative stress can induce degradation of basal membranes proteins by MMPs, which leads to BBB injury [37]. Albumin has been shown to bind to endothelial cells resulting in the activation of the TGF-β pathway [63]. However, the effects of albumin on the production of MMP-9 from other components of the neurovascular bundle remain to be determined.

Compromise of the BBB after TBI, stroke, or status epilepticus may expose the brain parenchyma to high molecular weight proteins from which it is normally protected. Of these proteins, both albumin and thrombin have been implicated in pathophysiologic processes including epileptogenesis [11,24] and intracerebral hemorrhage [16]. Acting through protease-activated receptor-1, thrombin activates MMP-9 in astrocytes [19], a mechanism linked to the pathogenesis of intracerebral hemorrhage after administration of tissue plasminogen for treatment of stroke [7]. *In vivo*, it is likely that the brain parenchyma is exposed to thrombin and albumin simultaneously with MMP-9, and studies are needed to investigate these responses, as has been previously carried out for the combined effects of thrombin and MMP-9 [36,64].

## Conclusions

In summary, these results link albumin acting through ROS and the p38 MAPK, to the activation of MMP-9 in astrocytes. Numerous studies identify a role for MMP-9 in the mechanisms of compromise of the BBB, epileptogenesis or synaptic remodeling after ischemia or TBI [9,10,12,14]. The increase in MMP-9 produced by albumin further implicates both astrocytes and albumin in the acute and long-term complications of acute CNS insults, including cerebral edema and epilepsy.

## Abbreviations

ANOVA, Analysis of variance; ALIAS, Albumin in Acute Stroke trial; BSA, Bovine serum albumin; carboxy-H2DCF-DA, carboxy-2′,7′-dichlorodihydrofluorescein diacetate; CNS, Central nervous system; CX3CL, C-X3-C motif ligand; DAPI, 4′,6-diamidino-2-phenylindole; DMEM, Dulbecco’s modified Eagle’s medium; DPI, Diphenyleneiodonium chloride; EDTA, Ethylenediamine tetraacetic acid; ELISA, Enzyme-linked immunosorbent assay; ERK, Extracellular signal regulated protein kinase; FBS, Fetal bovine serum; HBSS, Hank’s balanced buffered salt solution; JNK, c-Jun N-terminal kinase; IL, Interleukin; MAPK, Mitogen-activated protein kinase; MEK, Mitogen-activated protein kinase kinase; MCP, Monocyte chemotactic protein; NMDA, N-methyl-D-aspartate; ROS, Reactive oxygen species; PBS, Phosphate-buffered saline; PEG-CAT, Polyethylene glycol–catalase; PEG-SOD, Polyethylene glycol–superoxide dismutase; SAFE, Saline vs. Albumin Fluid Evaluation study; TBHP, Tert-butyl hydroperoxide; TBI, Traumatic brain injury; TGF, Transforming growth factor.

## Competing interests

The authors declare that they have no competing interests.

## Authors’ contributions

HRR co-conceived and designed the study; acquired, analyzed and interpreted data; and drafted the manuscript. JNH and NC acquired and analyzed data. MSW co-conceived and designed the study; analyzed and interpreted the data; and drafted the manuscript. All authors read and approved the final manuscript.
